# Prognostic Factors at Admission for In-Hospital Mortality from COVID-19 Infection in an Older Rural Population in Central Spain

**DOI:** 10.3390/jcm10020318

**Published:** 2021-01-16

**Authors:** Modesto M. Maestre-Muñiz, Ángel Arias, Laura Arias-González, Basilio Angulo-Lara, Alfredo J. Lucendo

**Affiliations:** 1 Department of Internal Medicine, Hospital General de Tomelloso, 13700 Ciudad Real, Spain; m4modesto@gmail.com (M.M.M.-M.); basiangulo@gmail.com (B.A.-L.); 2 Research Unit, Hospital General La Mancha Centro, Alcázar de San Juan, 13600 Ciudad Real, Spain; angel_arias_arias81@hotmail.com; 3 Centro de Investigación Biomédica en Red de Enfermedades Hepáticas y Digestivas (CIBERehd), Spain; 4 Department of Gastroenterology, Hospital General de Tomelloso, 13700 Ciudad Real, Spain; laura.arias.gonzalez@gmail.com; 5 Instituto de Investigación Sanitaria La Princesa, 28006 Madrid, Spain

**Keywords:** risk factors, predictors, mortality, COVID-19, Spain

## Abstract

Background: Risk factors for in-hospital mortality from severe coronavirus disease 2019 (COVID-19) infection have been identified in studies mainly carried out in urban-based teaching hospitals. However, there is little data for rural populations attending community hospitals during the first wave of the pandemic. Methods: A retrospective, single-center cohort study was undertaken among inpatients at a rural community hospital in Spain. Electronic medical records of the 444 patients (56.5% males) admitted due to severe SARS-CoV-2 infection during 26 February 2020–31 May 2020 were reviewed. Results: Mean age was 71.2 ± 14.6 years (rank 22–98), with 69.8% over 65. At least one comorbidity was present in 410 patients (92.3%), with chronic obstructive pulmonary disease (COPD) present in 21.7%. Overall in-hospital mortality was 32%. Multivariate analysis of factors associated with death identified patients’ age (with a cumulative effect per decade), COPD as a comorbidity, and respiratory insufficiency at the point of admission. No additional comorbid conditions proved significant. Among analytical values, increased serum creatinine, LDH > 500 mg/dL, thrombocytopenia (<150 × 10^9^/per L), and lymphopenia (<1000 cells/µL) were all independently associated with mortality during admission. Conclusions: Age remained the major determinant for COVID-19-caused mortality; COPD was the only comorbidity independently associated with in-hospital death, together with respiratory insufficiency and analytical markers at admission.

## 1. Introduction

The first cases of pneumonia caused by the novel coronavirus “severe acute respiratory syndrome coronavirus 2” or SARS-CoV-2 [1] were reported almost one year ago in Wuhan, China [2]. The disease caused by the virus has been named coronavirus disease 2019 (COVID-19) and has spread rapidly to affect more than 50 million people overall worldwide, with Spain being one of the most affected countries [3]. The several studies that have evaluated prognostic factors for mortality, including demographic, clinical, and analytical features, have demonstrated advanced age and comorbidity as major determinants for death. Other risk factors have varied widely from one study to another, most likely due to demographic and age-related differences among populations.

Despite the extensive literature on this topic, little data has been provided for rural populations attending community hospitals during the first wave of the COVID-19 pandemic, and it is not known whether risk factors associated with mortality in urban-based large teaching hospitals can be faithfully extrapolated to other environments.

Understanding the clinical risk factors and laboratory findings associated with severe and fatal COVID-19 among populations in a rural setting might allow early interventions to help mitigate adverse outcomes. The objective of this study is to identify risk factors for death from the COVID-19 infection among subjects admitted to a hospital in central Spain, and to analyze factors that may contribute to mortality.

## 2. Materials and Methods

We conducted a single-center, retrospective cohort study at a 160-bed community medical center located in a rural area of central Spain. The study protocol conforms to the ethical guidelines of the 1975 Helsinki Declaration of the World Medical Association on principles for medical research involving human subjects. The study was approved by the institutional research board at Mancha Centro General Hospital.

### 2.1. Study Population

The study cohort consisted of adult inpatients who were confirmed COVID-19 positive either by a nasopharyngeal swab test using real-time reverse-transcriptase-polymerase-chain-reaction (RT-PCR) assay, or by IgG/IgM lateral flow immunoassay chromatography rapid testing and who were admitted to hospital due to respiratory failure between 26 February 2020 and 31 May 2020.

Our hospital set up a commission in charge of reviewing the evidence on the prognosis and treatment measures for COVID-19. This commission developed and updated an institutional care protocol which guided the therapeutic decisions of physicians from different specialties to caring for patients admitted with COVID-19 under homogeneous criteria. The protocol gave advice on identifying clinical presentation of the infection, the tests to carry out on patients attending the emergency room, how to score patients according to severity criteria, and the recommended treatment options according to patients’ severity and prognosis. The intervals analysis and repeated chest X-rays were also recommended. Although the protocol included criteria for referral to the intensive care unit (ICU) of other hospitals in the region (as this resource is not present in our hospital), the serious restriction of ICU beds during the pandemic in the referral hospitals prevented the admission of all patients, from whom potential candidates could have been derived. Respiratory insufficiency was defined as an O_2_ saturation of less than 90% by pulse oximetry and/or a partial arterial pressure of oxygen (paO_2_) below 60 mmHg, both measured while breathing ambient air, with or without an increase in partial arterial pressure of CO_2_ (PaCO_2_) above 45 mmHg.

### 2.2. Data Source and Selection of Variables

Electronic medical records were reviewed for all the patients meeting the inclusion criteria. Data was anonymized before inclusion on an access database designed for the study. The outcomes analyzed were the in-hospital mortality rate and time to hospital discharge.

Data collected included demographic factors such as age, sex, normal place of residence (home or institutionalized), presence of co-morbid conditions (including cardiovascular, liver, respiratory and kidney diseases), life style, and immunosuppressant therapies. COVID-19 symptoms and time from start were considered. At admission vital signs, laboratory markers and radiographic findings were collected. Oxygen supply or any need of respiratory support, and all other therapies administered for COVID-19 were registered, including the need for ICU admission. Many of the variables used in this study were identified from previously published COVID-19 and critical care evidence.

### 2.3. Data Quality Assessment

The inclusion of data on the database was carried out in duplicate by two researchers with experience in the management of COVID-19. Disagreements were resolved after manually consulting the source data. Data completion was assessed based on the following three pivotal groups of variables: “demographic and clinical characteristics”, “COVID-19 management,” and “outcomes”. Duplicates were removed. Additionally, after data extraction and prior to statistical analysis, the database was reviewed for inconsistencies and subsequently subjected to data cleaning.

### 2.4. Statistical Analysis

The continuous variables with a normal distribution are presented as mean ± standard deviation (SD). Non-normally distributed continuous variables are reported as median ± interquartile range (IQR). Categorical variables are presented as percentages. Comparisons between continuous variables were performed by the Student *t*-test if they had a normal distribution, or otherwise by the Mann-Whitney U test. Comparisons between categorical variables were performed by the χ^2^ test or Fisher exact test when appropriate.

We performed separate forward stepwise logistic regression analyses in order to evaluate factors associated with death during hospital admission. In the model we included all variables significantly associated with death in the univariate analysis (*p* < 0.10), avoiding over adjustment, and potential determinants for unfavorable outcomes described in the literature.

Statistical processing of the data was performed with SPSS for windows, v.18.0; IBM Corp, Armonk, NY, USA. A *p* value < 0.05 was considered significant.

## 3. Results

**Figure 1:**
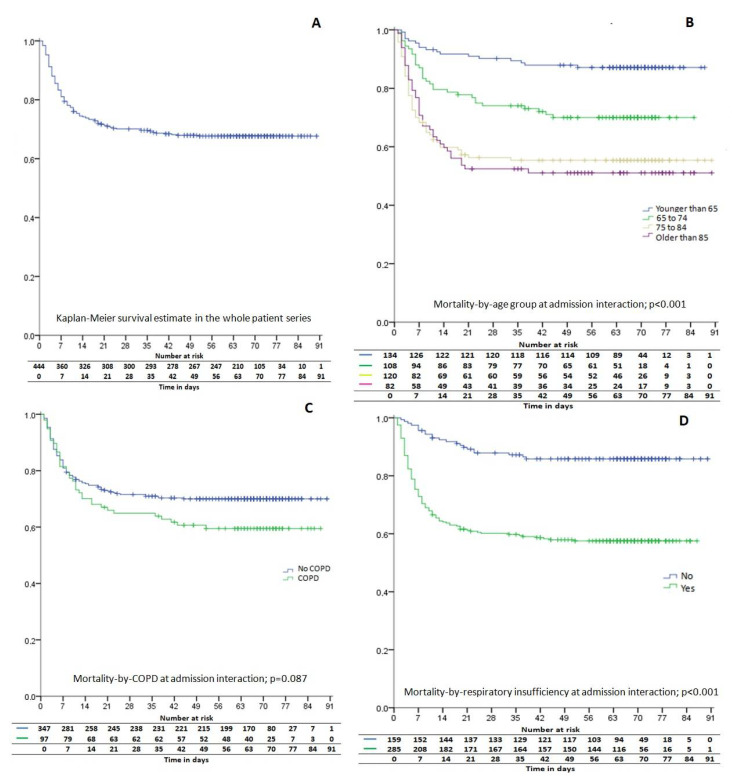
Kaplan-Meier survival estimate for patients admitted to our hospital due to severe coronavirus disease 2019 (COVID-19) in the first wave of the pandemic (**A**). Kaplan-Meier curve analysis for patient stratification by age group (**B**), comorbid chronic obstructive pulmonary disease (COPD) (**C**), and respiratory insufficiency (**D**) at admission. Statistical differences were assessed by Log Rank (Mantel-Cox).

During the study period, a total of 444 patients (56.5% of males) were admitted to our hospital. The clinical and demographic characteristics of the patients are presented in Table 1. Briefly, the mean age was 71.2 ± 14.6 years (rank 22–98), with 69.8% aged over 65. Overall in-hospital mortality was 32%, 12.7% in patients younger than 65 years, 29.6% in 65–74 years, 44.2% in patients 75–84 years, and 48.8% in persons older than 85 years. Patients who died were older (mean [SD] age, 77.4 [10.9] years vs. 68.2 [15.1] years; *p* < 0.001) than patients who survived. The Kaplan-Meier estimate for overall median survival was 25 days (95% confidence interval (CI), 15.6−34.4) (Figure 1). After 14 days from hospital admission, 74.55% (95% CI, 70.39–78.71) of our patients were still alive. At 28 days, the proportion of survivors in our series was 70.27% (95% CI, 65.91–74.63).

At least one comorbidity was present in 410 (92.3%) of the patients, the most common being hypertension (68.2%), obesity (46%), diabetes (32.2%), and chronic pulmonary disease (COPD/asthma) (27.7%). A higher mean (SD) number of co-morbidities was observed among those patients who died compared to those who survived, 3.6 [1.9] vs. 2.7 [1.8]; *p* < 0.001). The mean duration of symptoms prior to hospitalization was 7 (5.3; rank 0–37) days, and consisted of fever (64.4%), a cough (63.1%), and dyspnea (79.1%). Less frequently, the patients experienced diarrhea, nausea and vomiting, nasal congestion, and odynophagia. Pneumonia was diagnosed at admission in 413 (93%) patients. Seventy-one (16%) patients were admitted from nursing homes. Hospitalization time until discharge or death was 11.2 ± 10.3 days, being significantly longer in patients who were discharged compared to those who died (11.9 [10.4] vs. 9.8 [10.1] days; *p* = 0.045).

Thirty-five patients required noninvasive mechanical ventilation provided with pressure ventilation with continuous positive airway pressure (CPAP), and 28 underwent orotracheal intubation. Of the 28 patients, 25 were transferred to an ICU; 18 eventually died.

Laboratory findings at admission are presented in Table 2. Patients who died from COVID-19 infection had lower mean platelet count (×10^9^ per L [SD] 195.8 [74.3] vs. 231.4 [102.5]; *p* < 0.001), but similar white cells count. However, a trend towards a lower lymphocyte count (×10^6^ per L [SD] 945 [960] vs. 1100 [770]; *p* = 0.07) was observed among patients who died. Patients who died had significantly increased inflammatory response with significantly elevated C-reactive protein (CRP) levels (mean [SD] 164 [99] vs. 126 [102] mg/L; *p* < 0.0001), but no significant D-dimer or ferritin serum levels. Respiratory failure in the emergency room was worse among patients who died (partial pressure of oxygen—pO2 (mean SD) 55.7 [19.5] vs. 65.6 [26] mmHg, *p* < 0.001). In addition, these patients also had significantly more acute renal injury (serum creatinine levels (mean [SD] 1.66 [1.05] vs. 1.11 [0.5] mg/L; *p* < 0.0001), urea (76.5 [48.5] vs. 48.8 [32.4] mg/dL; *p* < 0.001), and heart injury (ultra-sensitive troponin (mean [SD] 134.6 [283.2] vs. 28.23 [45.3] ng/mL, *p* = 0.046) on admission compared to patients who survived, which may help explain the higher mortality rates. Lactate dehydrogenase (LDH) values were also significantly higher among patients who died (mean [SD] 838.7 [249.3] vs. 594.4 [193.1]; *p* < 0.001).

The treatments administered to patients are summarized in Table 3. A wide variety of drugs with a theoretical antiviral effect were used in 357 (80.4%) patients, the most frequent being hydroxychloroquine (351, 98.3%), and lopinavir/ritonavir (190, 53.2%). Remdesivir was used only in 4 patients (0.9%). Immunomodulatory drugs were also used frequently (134, 30.2%), mainly corticosteroids (134, 30.2%), but also tocilizumab (11, 8.2%), beta-interferon (5, 3.7%), anakimra (5, 3.7%) barizitinib (1, 0.2%), and ruxolinitib (3, 2.2%).

In 99 patients (22.3%), heparin of low molecular weight was used, either in prophylactic (43, [43.4%]), intermediate, or anticoagulant doses (80 [80.2%]).

### Factors Associated with Mortality at Hospital Admission

Multivariate logistic regression analysis to evaluate factors associated with in-hospital death identified patients’ age as the most relevant determinant, with a cumulative effect that increased with each decade of patient age). Specifically, patients aged 65–74 years presented an odds ratio (OR) for mortality of 3.04 in comparison to patients younger than 65; the OR rose to 4.22 among patients aged 75–84, and reached 8.16 among those over 85 years of age. Presenting chronic obstructive pulmonary disease (COPD) as a comorbidity increased the risk of death with an OR of 2.01, and suffering respiratory insufficiency at admission with an OR of 2.31.

Analytical values predicting mortality included creatinine concentration (each 1 mg/dL increase multiplying by 3 the risk of death; OR 3.12), high values (>500 U/L) of LDH (OR 4.61), thrombocytopenia (<150 × 10^9^ per L), (OR 2.84), and lymphopenia (<1000 cells/µL), (OR 1.75). Notably, suffering comorbid conditions other than COPD, nursing-home residency, immunosuppression, or an oncologic disease was not independently associated with overall risk of mortality (Table 4). Despite specific treatments being used more frequently in patients who survived severe COVID-19, no drug had a significant association with the outcome of the disease in our series of hospital admitted patients.

## 4. Discussion

A high mortality rate for COVID-19 infection, especially in the elderly, has already been demonstrated. In this study, we assessed the clinical characteristics at the admission of patients with more severe infections admitted to our hospital in the first wave of the COVID-19 pandemic. The overall proportion of deaths was 32%, much higher than that observed in China [4,5], similar to that reported in other areas of Spain [6,7,8] and neighboring European countries [9,10], and lower than that reported in United States [11], for the same age ranges. The threshold of severity required for hospitalization could explain some of these differences, as too could the incomplete follow up of patients assessed in some studies.

The risk of mortality in our cohort was independently associated with demographic factors, comorbidities, deterioration in clinical functions on admission, and certain analytical factors. Among the former, our study identified age over 65 as the most relevant independent risk factor for death due to severe COVID-19; the mean age of our cohort was 71.2 years, which contrasts with the average age of 66 years reported for patients overall suffering from COVID-19 admitted to hospital in Spain during the same period [12]. The age of our patients also exceeded that for the 15,111 patients included in a multicenter retrospective registry promoted by the Spanish Society of Internal Medicine (69.4 years) [6], that of the largest case series in New York (63 years) [11], and that reported in Wuhan of 56 years [13]. Studies repeatedly identified advanced age as a major risk factor for in-hospital mortality [11,13,14,15,16,17,18,19,20,21,22], and our results are close to those provided from additional series mainly involving elderly patients [13,14]. In our series, only 12.7% mortality occurred in patients under 65, and this was almost multiplied by 2 every 10 years up to more than 8 in patients over 85 years, further highlighting the association between age and fatal outcome.

In contrast, patients’ sex was not associated significantly to mortality. While male sex has been revealed as a risk factor for mortality in several studies carried out both in Spain [22] and other countries [11,14,15,18,23,24], it was not in many others [17,20,21,25,26,27]. Male sex could act, therefore, as a confounding variable for other risk factors more commonly described among males, such as cardiovascular diseases or tobacco use. Further studies should assess this hypothesis.

The vast majority of our patients presented one or more comorbidities, the most common of which were arterial hypertension (68.3%), obesity (46%), diabetes (32.2%), and COPD/asthma (27.7%). This prevalence, as well as for chronic kidney disease, chronic heart failure, and ischemic heart disease, were remarkably similar to those found in a general Spanish population aged over 65, according to the National Institute of Statistics or INE (INE Health Survey 2017). Bivariate analysis showed that almost all comorbidities were significantly more prevalent among patients who died, but only COPD was an independent risk factor in multivariate analysis. COPD was present in 22% of patients admitted to our hospital due to severe COVID-19, and in the 59.8% of patients who died. Considering that its prevalence in people over 45 in Spain is only 9.1% [28], this contributes to explaining that COPD by itself doubled the risk of mortality among our patients, once the other risk factors were controlled.

The relationship between risk of in-hospital mortality and other comorbidities proposed in some series, including high blood pressure [29], diabetes [16,20], or obesity [30,31,32,33] were not found in our study. This is in line with the results obtained by two meta-analyses carried out by Wu et al. [34] and Figliozzi et al. [24], which demonstrated that comorbidities and organ dysfunction can aggravate COVID-19, but not directly determine death. The high prevalence of all these comorbidities in people aged >65 years makes them more susceptible to developing severe COVID-19 and requiring hospital admission. In any case, overall mortality increased with the number of comorbidities in bivariate analysis, in agreement with results of large descriptive series attended during the first wave of the pandemic [27].

Most of our patients were severely ill, and a large proportion needed supplemental oxygen upon admission. A relationship between partial arterial pressure of oxygen at admission and mortality was found, thus indicating that respiratory insufficiency on admission predicts mortality. As already reported in previous series, most of our patients (91%) presented pneumonia at admission, more commonly bilateral pneumonia (80.6%) [8,23,35,36], with typical infiltrates associated with SARS-Cov-2 pneumonia. Severity of pneumonia has been recognized as an independent risk factor for mortality or ICU admission [22,23,37]. In contrast, mortality was also high (29%) and not significantly different among the 35 patients (8%) who did not present pneumonia upon admission. The low number of patients in this group prevents us from analyzing additional reasons for this inconsistency with previous literature.

As for the analytical findings, bivariate analyses identified thrombocytopenia, lymphopenia and elevated serum LDH, CRP, ultrasensitive Troponin I, urea, and creatinine as significantly more common in deceased patients. All of these afflictions have been shown to be related to severity [13,14] or independently associated with risk of death due to COVID-19 [17,18,23], and also specifically among Spanish patients [22,26,36]. Conversely, we did not find any relationship between D-dimer, WBC, or ferritin levels and mortality, as other studies did [13,14,23,24], nor among Spanish patients [22,26]. The fact that only a minority of patients had some of these values available at admission provides a suitable explanation for this. After multivariate analysis, increase serum creatinine >1.1 mg/dL (with each additional 1.0 mg increase raising the OR of death 3.2 times) and LDH >500 IU/L values were revealed as independent predictors of mortality, together with thrombocytopenia <150,000/µL and lymphopenia <1000 cells/µL, thus confirming previous observations associating severe COVID-19 infection with decreased number of platelets [38] and lymphocytes [39,40]. Whether cytopenias results in disease severity or the severity of COVID-19 decreases cells counts is still open to debate.

Our study also found an inverse relationship between time from symptom onset to hospital admission and mortality risk. That is to say the correlation rate was 20% among patients who were admitted within a week of disease onset, compared to 37.4% in those who were admitted later. This trend persisted even when lower latency cut-offs were considered. In fact, a shorter time from disease onset has been identified as an independent risk factor for COVID-19-related mortality by additional authors [19,41], although this finding has not been universally reproduced [19]. In any case, it is likely that a more abrupt and intense onset of symptoms indicates a more serious disease and therefore the need for earlier medical contact, facilitating the establishment of an early treatment that could improve the prognosis.

Delirium on admission has been previously identified as a predictor for mortality in patients with COVID-19 [42,43]; a 59% mortality rate was also documented among patients who experienced delirium on admission at our hospital. However, after multivariate analysis, delirium was found not to independently determine mortality, most likely due to the fact that it involves acute failure of brain processes and is strongly related to hypoxemia in critical ill patients.

Only 6.8% of our patients were admitted to ICU during the study period, which contrasts with 17% admission rates reported in earlier Chinese reports [13] and 30.8% rates reported at rural hospitals in the USA [25]. ICU admission rates in other Spanish hospitals were 13% [22]; however, mortality was high, and similar to the rates which we found in our series (72%). Low admission rates to ICU are explained by unavailability of beds in our referral hospitals during the first wave of the pandemic and absence of agreements, initially, for effective referral of patients to other health areas.

A wide variety of drugs with potential antiviral effects and immunomodulatory agents were used to treat our patients, together with supporting measures. However, in the results provided by multivariate analysis, none of these drugs provided evidence of a significant impact on the prognosis of patients, despite all of them being used more intensively among those patients who survived. Treatments, including more aggressive support measures beyond the effect of the specific drugs used, would explain these differences in the results.

The limitations of our study include its observational design, which prevented us from establishing cause-effect relationships, and the retrospective acquisition of data, which were obtained from a single site providing services to a rural population and therefore might not be extrapolated to other environments. While study data was generated at a time of healthcare crisis, some variables of potential interest may not have been collected. Finally, only in-hospital mortality was measured as a primary outcome, and therefore no further follow-up of patients was provided for analysis. Considering the age and frequent comorbidities of our patients, with some being transferred to nursing homes after discharge, the true mortality attributed to COVID-19-related complications could be greater. Despite these limitations, our study also has notable strengths, including the exhaustive inclusion of all consecutive patients with severe COVID-19 who were admitted to hospital, the use of standardized protocols to manage patients’ profiles at our institution and the measures we took to ensure data quality.

In summary, we provide evidence here of high in-hospital mortality caused by COVID-19. Risk factors identified largely reproduce those already reported in previous series recruited at urban-based and teaching hospitals, with advance age being the major determinant for a poor prognosis. High mortality was attributed to an older population with chronic comorbidities, with COPD being especially relevant. Respiratory insufficiency and some analytical markers at admission were independently associated with mortality risk. Older age, clinical, and laboratory findings should alert healthcare providers to identify patients at the highest risk for severe COVID-19 associated outcomes. Subsequent research, especially data provided by patients admitted during the second wave of the pandemic, may validate our findings and aid early clinical decision making.

## Figures and Tables

**Table 1:** Demographic, baseline comorbidities and clinical characteristics at admission of a series of patients admitted to our due to COVID-19, and differences between patients who survived and dead. COPD: chronic obstructive pulmonary disease; CKD: chronic kidney disease; CHF: congestive heart failure; FIO2: fraction of inspired oxygen.

			Total (*n* = 444)	Survivors (*n* = 302)	Dead (*n* = 142)	*p*
Mean age at admission, years (SD; rank)			71.2 (14.6; 22–98)	68.2 (15.1)	77.4 (10.9)	**<0.001**
	bellow 65, *n* (%)		134 (30.2%)	117 (87.3%)	17 (12.7%)	**<0.001**
	65 to 74, *n* (%)		108 (24.3%)	76 (70.4%)	32 (29.6%)	
	75 to 84, *n* (%)		120 (27%)	67 (55.8%)	53 (44.2%)	
	over 85, *n* (%)		82 (18.5%)	42 (51.2%)	40 (48.8%)	
Sex	Male, *n* (%)		251 (56.5%)	172 (68.5%)	79 (31.5%)	0.794
	Female, *n* (%)		193 (43.5%)	130 (67.4%)	63 (32.6%)	
Smoker	No, *n* (%)		317 (71.4%)	210 (66.2%)	107 (33.8%)	0.296
	Yes, *n* (%)		21 (4.7%)	17 (81%)	4 (19%)	
	Former, *n* (%)		106 (23.9%)	75 (70.8%)	31 (29.2%)	
BMI	Normal weight, *n* (%)		138 (31.1%)	97 (70.3%)	41 (29.7%)	0.057
	Overweight, *n* (%)		102 (23%)	73 (71.6%)	29 (28.4%)	
	Obesity class 1, *n* (%)		110 (24.8%)	66 (60%)	44 (40%)	
	Obesity class 2, *n* (%)		74 (16.7%)	56 (75.7%)	18 (24.3%)	
	Obesity class 3, *n* (%)		20 (4.5%)	10 (50%)	10 (50%)	
Other comorbidities	Immunosuppression, *n* (%)	Yes	26 (5.9%)	14 (53.8%)	12 (46.2%)	0.110
		No	418 (94.1%)	288 (68.9%)	130 (31.1%)	
	Arterial hypertension, *n* (%)	Yes	303 (68.2%)	194 (64%)	109 (36%)	**0.008**
		No	141 (31.8%)	108 (76.6%)	33 (23.4%)	
	Diabetes, *n* (%)	Yes	143 (32.2%)	94 (65.7%)	49 (34.3%)	0.477
		No	301 (67.8%)	208 (69.1%)	93 (30.9%)	
	Hypothyroidism, *n* (%)	Yes	58 (13.1%)	37 (63.8%)	21 (36.2%)	0.459
		No	386 (86.9%)	265 (68.7%)	121 (31.3%)	
	Oncological disease, *n* (%)	Yes	45 (10.1%)	25 (55.6%)	20 (44.4%)	0.059
		No	399 (89.8%)	277 (69.4%)	122 (30.6%)	
	Asthma, *n* (%)	Yes	31 (7%)	22 (71%)	9 (29%)	0.715
		No	413 (93%)	280 (67.8%)	133 (32.2%)	
	COPD, *n* (%)	Yes	97 (21.8%)	58 (59.8%)	39 (40.2%)	**0.049**
		No	347 (78.2%)	244 (70.3%)	103 (29.7%)	
	CKD > stage II, *n* (%)	Yes	59 (13.3%)	31 (52.5%)	28 (47.5%)	**0.006**
		No	385 (86.7%)	271 (70.4%)	114 (29.6%)	
	CHF > Class II, *n* (%)	Yes	57 (12.8%)	30 (52.6%)	27 (47.4%)	**0.008**
		No	387 (87.2%)	272 (70.3%)	115 (29.7%)	
	Ischemic heart disease, *n* (%)	Yes	57 (12.8%)	32 (56.1%)	25 (43.9%)	**0.039**
		No	387 (87.2%)	270 (69.8%)	117 (30.2%)	
	Chronic liver disease, *n* (%)	Yes	31 (7%)	19 (61.3%)	12 (38.7%)	0.405
		No	413 (93%)	283 (68.5%)	130 (31.5%)	
Mean (SD) number of comorbidities, *n* (%)			3.1 (1.9)	2.7 (1.8)	3.6 (1.9)	**<0.001**
Nursing-home residents, *n* (%)		Yes	71 (16%)	40 (56.3%)	31 (43.7%)	**0.021**
		No	373 (84%)	262 (70.2%)	111 (29.8%)	
Presenting Symptoms	Dyspnea, *n* (%)		351 (79.1%)	229 (65.2%)	122 (34.8%)	**0.015**
	Cough, *n* (%)		280 (63.1%)	204 (72.9%)	76 (27.1%)	**0.004**
	Fever, *n* (%)		286 (64.4%)	202 (70.6%)	84 (29.4%)	0.112
Days from first symptoms to admission, mean (SD)			7 (5.3; 0–37)	5.37 (4.08)	7.76 (5.65)	**<0.001**
Respiratory insufficiency		Yes	285 (64.2%)	165 (57.9%)	120 (42.1%)	**<0.001**
		No	159 (35.8%)	137 (86.2%)	22 (13.8%)	
FIO2 to maintain O2 saturation >90%, mean (SD, rank)			42.4 (30.9; 21–100)	36.27 (26.3)	55.37 (35.6)	**<0.001**
Pneumonia	Unilateral		51 (11.55)	39 (76.5%)	12 (23.5%)	0.238
	Bilateral		358 (80.6%)	237 (66.2%)	121 (33.8%)	
	Lobar		4 (0.9%)	4 (100%)	0	
Mean (SD) days from admission to discharge or dead			11.2 (10.3)	11.9 (10.4)	9.8 (10.1)	**0.045**

Figures marked in bold indicate a statistically significant difference (*p* < 0.05).

**Table 2:** Laboratory values, expressed as mean ± standard deviation, at admission of patients with COVID-19 overall, and in those who survived or died.

	Total (*n* = 444) *N* (%)	Survivors (*n* = 302) *N* (%)	Dead (*n* = 142) *N* (%)	*p*
Hemoglobin, g/dL	13.6 (1.9)	13.8 (1.8)	13.3 (2)	**0.018**
Leukocytes, ×10^6^, per L	8326 (3866.2)	8223.4 (3646.8)	8544.5 (4301.8)	0.444
Lymphocytes, ×10^6^, per L	1050.5 (839.4)	1099.4 (775.9)	945.7 (956.2)	0.074
Platelets, ×10^9^, per L	219.84 (96.09)	231.40 (102.50)	195.80 (74.28)	**<0.001**
Fibrinogen (*n* = 183)	636 (179)	628.7 (187.8)	654.4 (154.7)	0.382
D Dimer, ng/mL (*n* = 266)	2.8 (4.4)	2.75 (4.4)	3.03 (4.4)	0.650
C-reactive protein, mg/L	138 (102)	126 (102)	164 (99)	**<0.001**
Urea, mg/dL	57.7 (40.4)	48.8 (32.4)	76.5 (48.5)	**<0.001**
Creatinine, mg/dL	1.29 (0.8)	1.11 (0.5)	1.66 (1.05)	**<0.001**
AST, U/L (*n* = 331)	42.3 (28.4)	40.7 (27)	46.2 (31.6)	0.117
ALT, U/L (*n* = 351)	35.5 (33.7)	37.6 (37.6)	30.4 (20.6)	**0.023**
GGT, U/L (*n* = 96)	80.3 (107.5)	84.1 (109.6)	65.9 (100.8)	0.506
LDH, U/L (*n* = 337)	651.49 (275.24)	594.5 (246.70)	825.78 (286.07)	**<0.001**
Ferritin, ng/mL (*n* = 91)	1042.3 (770.3)	1043.8 (799.6)	1036 (658.2)	0.970
TSH, mU/L (*n* = 78)	1.2 (1.2)	1.25 (1.37)	1.06 (0.7)	0.569
T4 libre, μg/dL (*n* = 27)	1.3 (0.3)	1.36 (0.4)	1.29 (0.19)	0.643
Ultra-sensitive troponin, ng/dL (*n* = 87)	66.1 (178.7)	28.23 (45.3)	134.6 (283.2)	**0.046**
Interleukine 6, pg/mL (*n* = 19)	170.8 (302.3)	81.8 (75.6)	420.1 (535.9)	0.231
Partial arterial pressure of oxygen (paO2), mmHg	62.6 (24.6)	65.6 (26)	55.7 (19.5)	**<0.001**
Partial arterial pressure of carbon dioxide (paCO2), mmHg	35.5 (7.6)	35 (6.8)	36.6 (9)	0.077
HCO3, mEq/L	23.7 (4.1)	23.9 (3.7)	3.7)	0.122
Lactate, mmol/L	1.87 (4.5)	1.8 (5.3)	2 (1.4)	0.753

Figures marked in bold indicate a statistically significant difference (*p* < 0.05).

**Table 3:** Specific drug treatments used in patients hospitalized by COVID-19 at our site, and differences among patients who survived and died.

		Total (*n* = 444)	Survivors (*n* = 302)	Dead (*n* = 142)	*p*
No treatment, *n* (%)	Yes	**74 (16.7%)**	25 (33.8%)	49 (66.2%)	**<0.001**
	No	**370 (83.3%)**	277 (74.9%)	93 (25.1%)	
Antiviral drugs, *n* (%)	Yes	**357 (80.4%)**	269 (75.4%)	88 (24.6%)	**<0.001**
	No	**87 (19.6%)**	33 (37.9%)	54 (62.1%)	
Corticosteroids, *n* (%)	Yes	**134 (30.2%)**	111 (82.8%)	23 (17.2%)	**<0.001**
	No	**310 (69.8%)**	191 (61.6%)	119 (38.4%)	
Doxycyclin and/or acetylcysteine, *n* (%)	Yes	**41 (9.2%)**	38 (92.7%)	3 (7.3%)	**<0.001**
	No	**403 (90.8%)**	264 (65.5%)	139 (34.5%)	
Heparins of low molecular weight, *n* (%)	Yes	**99 (22.3%)**	74 (74.7%)	25 (25.3%)	0.103
	No	**345 (77.7%)**	228 (66.1%)	117 (33.9%)	

Figures marked in bold indicate a statistically significant difference (*p* < 0.05).

**Table 4:** Logistic regression analysis of factors associated with mortality, at the moment of hospital admission of patient with COVID-19. Respiratory insufficiency was defined as O2 saturation < 90% by pulse oximetry and/or a paO2 < 60 mmHg, both measured while breathing ambient air.

Predictor		Odds Ratio (95% Confidence Interval)	*p*
Age (years)	Below 65	Ref	-
	65 to 74	3.04 (1.21–7.66)	0.019
	75 to 84	4.22 (1.67–10.66)	0.002
	Over 85	8.16 (2.91–22.86)	<0.001
Chronic Obstructive Pulmonary Disease (COPD)		2.01 (1.01–4.02)	0.048
Respiratory insufficiency at admission		2.31 (1.16–4.62)	0.018
Analytical values at admission	Creatinine (each 1 mg/dL increase)	3.12 (1.95–5.01)	<0.001
	LDH (>500 U/L)	4.61 (1.95–10.91)	<0.001
	Platelets (<150 × 10^9^, per L)	2.84 (1.39–5.79)	0.004
	Lymphocytes (<1000 cells per µL)	1.75 (0.94–3.25)	0.080
